# Simple Shared Motifs (SSM) in conserved region of promoters: a new approach to identify co-regulation patterns

**DOI:** 10.1186/1471-2105-12-365

**Published:** 2011-09-12

**Authors:** Jérémy Gruel, Michel LeBorgne, Nolwenn LeMeur, Nathalie Théret

**Affiliations:** 1 EA 4427 SeRAIC IFR140, Université de Rennes 1, 2 avenue du Pr. Léon Bernard, Rennes, 35043, France; 2 IRISA, Université de Rennes 1, 263 avenue du Général Leclerc, Rennes, 35042, France

## Abstract

**Background:**

Regulation of gene expression plays a pivotal role in cellular functions. However, understanding the dynamics of transcription remains a challenging task. A host of computational approaches have been developed to identify regulatory motifs, mainly based on the recognition of DNA sequences for transcription factor binding sites. Recent integration of additional data from genomic analyses or phylogenetic footprinting has significantly improved these methods.

**Results:**

Here, we propose a different approach based on the compilation of Simple Shared Motifs (SSM), groups of sequences defined by their length and similarity and present in conserved sequences of gene promoters. We developed an original algorithm to search and count SSM in pairs of genes. An exceptional number of SSM is considered as a common regulatory pattern. The SSM approach is applied to a sample set of genes and validated using functional gene-set enrichment analyses. We demonstrate that the SSM approach selects genes that are over-represented in specific biological categories (Ontology and Pathways) and are enriched in co-expressed genes. Finally we show that genes co-expressed in the same tissue or involved in the same biological pathway have increased SSM values.

**Conclusions:**

Using unbiased clustering of genes, Simple Shared Motifs analysis constitutes an original contribution to provide a clearer definition of expression networks.

## Background

A major challenge for modern molecular biology consists in deciphering the complex regulation of gene expression. During the two last decades, numerous experimental and computational approaches have been developed to identify functional regulatory domains in genes. Binding sites for transcription factors (TFBS) are central elements in the modulation of transcriptional activity. These short DNA sequences are *cis*-regulatory motifs usually located in the proximal promoter region of target genes and bind trans-acting transcription factors [1]. Transcription factors have been shown to act cooperatively [2], leading to the emergence of the CRM (*cis*-regulatory modules) concept.

*In silico *approaches designed to uncover regulatory elements in gene promoters are based on this understanding of gene regulation. A possible binary categorization of these approaches is based on the fact that they use or not previously described TFBS, stored in databases such as TRANSFAC [3] or JASPAR [4]. Both strategies have advantages and drawbacks.

Although TFBS databases are admittedly incomplete, storing a small subset of the TFBS predicted to operate in eukaryotic genomes [5], searching for previously described TFBS is a much easier task than discovering *de novo *motifs [6]. Multiple tools have been following this approach working either on sets of genes [7-10] or designed to scan whole genomes [11-14]. The earlier methods were focusing on single TFBS, the more recently published mainly focus on CRM detection.

On one hand, not using TFBS databases allows not to be restrained by the yet incomplete set of described binding sites. On the other hand, uncovering regulatory elements becomes a much more challenging problem. Aiming at *de novo *discovering either single TFBS or CRM, such approaches are limited to sets of genes thought to be co-regulated [15-18] (for instance observed co-expressed) and have not yet been applied to whole genome experiments.

Aside from the CRM concept, the main advance in the field came from the accumulation of high-throughput gene expression data and the sequencing of multiple genomes. Multiple genome comparisons allows to select genetic regions undergoing a strong selection pressure and thus accumulating less mutations over time than the rest of a genome. Applied to promoter sequences, phylogenetic footprinting or phylogenetic shadowing allow to specify functional elements of the sequence, thus reducing the search space for transcription factors binding sites and the rate of false positive detected TFBS [19,20]. Most of the recently published algorithms make use of phylogenetic footprints instead of raw genomic data.

In the present study we propose a novel approach allowing genomewide regulatory element based searches without the need to rely on TFBS databases. Our main input dataset is the evolutionary conserved sequences of promoters obtained from the cisRED database [21]. Instead of using TFBS databases to narrow down the search scope, a gene of interest is selected. The methodology compares its evolutionary conserved sequences with those from all the other genes from cisRED, searching for statistically overrepresented sets of shared sequences. Although not permitting a straightforward extraction of the motifs involved, our study combines the advantages of performing genomewide searches and not being limited by described TFBS in order to find genes potentially co-regulated with a gene of interest.

The methodology is based on the compilation of statistically exceptional number of short, degenerate and shared sequences between gene pairs. We hypothesize that regulation of gene expression might be characterized by sequences involved in expression regulation whose common feature is to be evolutionary conserved and present in the promoter of genes (including *cis*-regulatory modules and potentially structural features or epigenetic patterns). Genes co-expressed should share some of these sequence features. We designed an algorithm taking as input the atomic motifs described in the cisRED database. Atomic motifs are evolutionary conserved sequences identified in the promoter of genes through a comparative analysis including more than 40 vertebrate species and making use of various motif discovery algorithms such as MEME [22], Consensus [8] or Motif Sampler [23]. The algorithm finds all Simple Shared Motifs (SSM), *i.e. *sets of complementary reverse sequences defined by their length *l*, Hamming edition distance *d *[24], and their occurrence in gene pairs. The number of SSM is then statistically assessed and groups of genes with an exceptional number of SSM are compiled. This simple methodology allows to perform a genomewide search for genes potentially co-regulated with a gene of interest by selecting a set of genes (out of the 18000 genes provided in the cisRED database) sharing a statistically exceptional SSM profile with the given gene. In support of our approach, we carried out a functional analysis of identified genes by using gene-set enrichment analysis (GSEA) [25,26] and gene-expression meta-analysis (Gemma [27]). Using Gene Ontology and KEGG pathways annotation ([28] and [29]), we demonstrate that the genes identified by our SSM approach are overrepresented in specific biological categories. We further show that these genes are more often co-expressed in expression array databases than randomly selected genes, thus suggesting that the SSM approach identifies genes that share common regulatory mechanisms. As a reverse experiment, we applied the SSM analysis to genes previously reported as belonging to the same biological pathway or co-expressed in the same tissue. We demonstrate that these genes contain significantly more SSM than genes chosen in different pathways or tissues, strengthening the association between SSM and regulatory patterns. We believe that SSM, as a set of composite conserved sequences, introduces a new concept in the identification of genes subject to similar patterns of regulation within a genome.

## Methods

### Simple Shared Motifs

Simple Shared Motifs (SSM) are sets of subsequences identified through a comparative analysis of atomic motifs from the cisRED database that contains more than 18,000 single human genes with 12.7 ± 8.9 atomic motifs per gene and a mean length of 11.7 ± 4.1 nucleotides per atomic motif. For each nucleotide sequence, we associate extended sequences as a set {*w*, *w_c_*, *w_r_*, *w_rc_*} where *w_c _*is the complementary sequence of *w*, *w_r _*its reverse sequence and *w_rc _*its reverse complementary sequence. If *d *is the standard Hamming distance between two sequences of same length, the distance between two extended sequences *W *and *W*' is *min*(*d*(*w*, *w*')) for all *w*, *w*' in *W *and *W*', respectively.

A SSM is a set of extended sequences extracted from the atomic motifs from of a gene pair (g1,g2) and parameterized using two integers (*l*, *d*). *l *is the length of the extended sequences in the SSM and *d *is the maximum distance between two extended sequences. A SSM must satisfy the three following properties:

1. the SSM contains at least an extended sequence coming from an atomic motif of g1 and an extended sequence coming from an atomic motif of g2;

2. the Hamming distance between two extended sequences of the SSM is at most *d*;

3. the set is maximal when these properties are fulfilled.

The last condition means that it is impossible to add any more sequences in the set while preserving conditions (1) and (2).

We designed an algorithm to build all (*l*, *d*)SSMs for a gene pair (g1,g2) (Additional file 1). We gather all the length *l *subsequences of atomic motifs in g1 and g2. We associate to each extended subsequence s, the set of genes *G*(*s*) containing *s*. Given a distance *d*, we build the graph where the *i *nodes are the *i *extended subsequences *s_i_*. An edge between *s*_1 _and *s*_2 _means that *d*(*s*_1_, *s*_2_) ≤ *d*. We compute all maximal cliques of the graph and obtain sets of extended sequences that satisfy properties 2 and 3. We finally discard all sets that do not satisfy property 1, the remaining set of subsequences are (*l*, *d*)SSMs. Our strategy is reminiscent of the one presented in [30].

To take into account the SSM occurrences arising by chance, we have to consider two factors: first, the number of subsequences in atomic motifs which increases with the length of atomic motifs; second, the probability of finding a subsequence by chance which increases with the distance parameter. As an example, let us take a search for (8, *d*)SSM. A gene g1 has two atomic motifs of length 9 and 10; the atomic motif of length 9 has two subsequences of length 8 while the atomic motif of length 10 has three. A gene g2 with only one atomic motif of length 10, has three subsequences of length 8. The total number of potential (8, *d*)SSM for the gene pair (g1,g2) is (2 + 3) × 3 = 15 subsequences. Considering a gene g3 with two atomic motifs of length 8 and 9 respectively, the total number of potential (8, *d*)SSM for the gene pair (g2,g3) is 3 × (1 + 2) = 9, or more generally, the product of the number of possible subsequences of length *l *in each gene set of atomic motifs. This suggests that small changes in the atomic motifs size can induced a broad variation in the potential number of SSMs. In order to correct for the influence of this noise in SSM counts, we use the ratio $SSMC=\frac{number\;of\;SSMs}{number\;of\;potential\;SSMs}$. Given a pair of genes *g*^1^, *g*^2^, both present in the cisRED database, we define SSMC(*g*_1_, *g*_2_, *l*, *d*) as the corrected count of (*l*, *d*)SSMs for the set of genes and hypothesize that an exceptionally high value is the mark of common regulation.

To measure the exceptional nature of the SSMC obtained for a pair of genes and a SSM type, defined as any SSM with given *l *and *d *parameters, we test the null hypothesis that the selected pair of genes has a higher SSMC than expected by chance with a random pair of genes. To do this, the distribution of SSMC for the whole set of cisRED genes is estimated through the analysis of the 50,000 pairs of randomly selected genes. This estimation is in the form of a cumulative distribution function which gives directly, for each SSMC value, the probability of finding an equal or greater SSMC value. This probability is used as an estimated *p*-value for the null hypothesis. A (*g*_1_, *g*_2_, *l*, *d*)SSMC value is considered exceptional if its estimated *p*-value is less than a defined threshold *t*. To capture the most exceptional number of SSMs independently of the length and edition distance, we introduce *cp*-values. Given a pair of genes *g*_1_, *g*_2 _and a list L of (*l*, *d*)*SSMs*, we define the *cp*-value as the lowest *p*-value among the *p*-values computed for *g*_1_, *g*_2 _and each of the (*l*, *d*)*SSMs*. A *cp*-value is considered exceptional if its value is less than a defined threshold *t*. Thus our method identifies lists of genes sharing a Combined EXceptional (CEX) number of SSMs, independently of the SSM type. Given a gene *g*, a list *L *of (*l*, *d*) SSMs and a *cp*-value threshold *t*, we define the *CEXlist*(*g*, *L*, *t*) as a subset of the cisRED genes. The CEXlist holds every cisRED gene that, paired with *g*, shows a *cp*-value below the defined threshold *t*.

### Functional analysis

**Figure 1:**
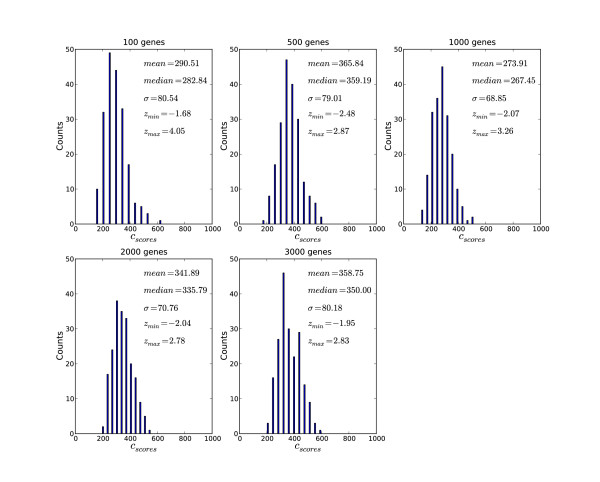
***c_score _*distribution of 200 groups of random gene pairs**. Each panel indicates the *c_score _*mean, median, standard deviation (*σ*) and the minimal and maximal obtained *z_scores _*(*z_min _*and *z_max_*).

To characterize the biological relevance of the CEXlists, we carried out a functional analysis by using gene annotations including Gene Set Enrichment Analysis (GSEA) [25,26] and gene expression data. The GSEA method consists in determining whether a defined set of genes shows an over-representation of biological annotations or categories such as Gene Ontologies or KEGG pathways. To perform this analysis, we used the Category and GOstats R packages that implement an improved GSEA [31] and are freely distributed on the Bioconductor project web site http://bioconductor.org/. Each gene from a selected list and the whole set of cisRED human genes were annotated with their category term and a hypergeometric test was computed to assess whether the number of selected genes associated with the term was greater than expected by chance. We defined the following score to measure the relative importance of gene over-representation in biological categories:

$$c_{score}=\overset{n}{\underset{i=0}{\sum }}\log\left(\frac{1}{p_{i}}-19\right)$$

where *p_i _*is the *p*-value associated with the *i^th ^*category among the n over-represented categories found for a list of genes; 19 is a constant chosen to give a *c_score _*= 0 for a category with *p*-value = 0.05 (threshold set of the hypergeometric test). This allows taking into account the number of over-represented categories together with the ranking of this over-representation (*i.e.*, *p*-value). We next estimated the *c_scores _*null distributions for lists of different sizes including 100, 500, 1000, 2000 and 3000 genes randomly selected from the cisRED database. Figure 1 displays the *c_scores _*null distributions and shows that they approximate a normal law. To assess the importance of a *c_score _*for a given list, we computed a standard *z_score _*:

$$z_{score}=\frac{c_{score}-\mu }{\sigma }$$

where *μ *and *σ *are the mean and standard deviation of the associated distribution, respectively. For a given list, a null *z_score _*implies that the over-representation of genes in biological categories equals the average representation of randomly selected genes, while a *z_score _*≥ 0 indicates an increase of gene clustering in categories.

Next, we carried out a comparative analysis between genes identified by our SSM approach and gene co-expression. For that purpose we used Gemma, a database containing hundreds of microarray datasets, and software that uses as input a gene of interest to generate a list of genes co-expressed in microarray experiments [27]. To compare CEXlists and Gemma lists, we computed the intersection according to different *cp-*values. The number of Gemma genes found in a CEXlist per gene belonging to the CEXlist is defined as a *density*:

$$density=\frac{\left|G\cap S\right|}{\left|S\right|}$$

where *G *is the set of genes obtained from Gemma and S is the set of genes from CEXlist. The significance of an enrichment in co-expressed genes in CEXlists was assessed by comparing the counts of Gemma genes per gene in the CEXlist to the counts of Gemma genes per gene out of the CEXlist using a standard Fisher test.

### Databases and biological resources

**CisRED: **the *cis*-REgulatory Database is a database for conserved regulatory motifs predicted in promoter regions http://www.cisred.org/. This study focuses on the atomic motifs extracted from the database, defined as: "a set of sequences, typically with a common length between 6 and 12 bp, members of which are present in a sequence region on the target species and in corresponding regions on other genomes" [21]

**GO: **the Gene Ontology database describes gene products in a species-independent manner by using three structured controlled vocabularies for biological processes, cellular components and molecular functions http://www.geneontology.org/.

**KEGG: **the Kyoto Encyclopedia of Genes and Genomes database is an integrated resource consisting of 16 main databases that include the KEGG Pathway for Metabolic and Signaling Pathways and KEGG Brite for Gene Ontology http://www.genome.jp/kegg/.

**TIGER: **the Tissue-specific Gene Expression and Regulation database contains tissue-specific expression profiles for 20,000 UniGene genes http://bioinfo.wilmer.jhu.edu/tiger/.

**Gemma: **Gemma is a database and software system for the meta-analysis of gene expression data, it contains data from hundreds of public microarray data sets http://www.chibi.ubc.ca/Gemma/.

**SSM types: **according to the size distribution for classical regulatory motifs, we selected SSMs with *l *ranging form 6 to 14. To avoid alignments due to pure chance, the editing distance *d *ranges from 0 to 5 and is no longer than a third of the SSM size.

## Results

### From SSMs determination to CEXlists computing

**Figure 2:**
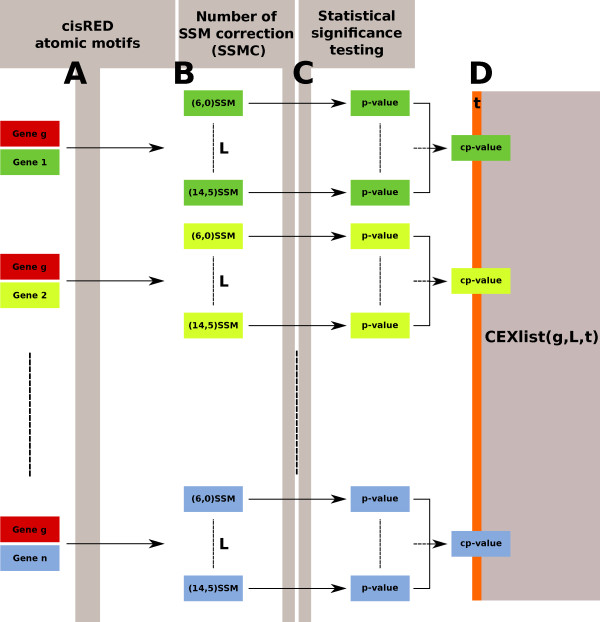
**Overview of the workflow leading to CEXlists**. A) The atomic motifs are extracted from the cisRED database for gene pairs associating the gene of interest *g *and the 18.000 other genes described in the database. B) The number of SSMs is computed for all the studied SSM types (*L*) and all the gene pairs. C) The numbers of SSMs are corrected by the amount of potential SSMs (SSMC) and the p-value testing the null hypothesis: "the SSMC value is not greater than expected by chance" is computed. D) The lowest *p*-value obtained for each gene pair is used as the *cp*-value. The CEXlist(*g*, *L*, *t*) retains the genes for which a *cp*-value below a threshold *t *is computed when paired with *g*.

**Figure 3:**
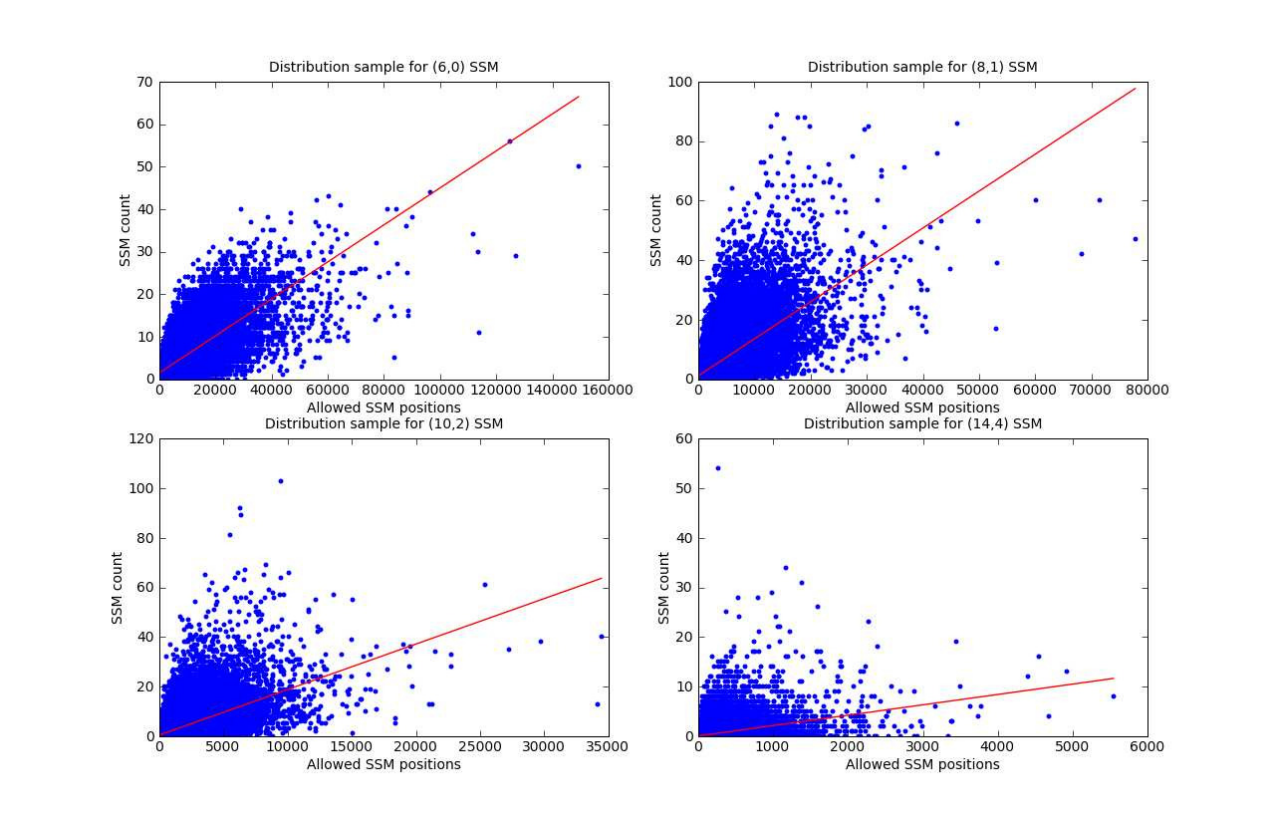
**Analysis of the correlation between the number of SSMs and the number of potential SSMs**. SSMs were identified for 50,000 pairs of randomly selected genes. Results are presented for 4 SSM types: (6,0)SSM; (8,1)SSM; (10,2)SSM and (14,4)SSM.

The overall workflow of the SSM based approach is described in Figure 2. Search for genes sharing similar motif pattern with a gene g1 consists in counting all (*l*, *d*)SSMs, *l *ranging from 6 to 14 and d from 0 to 5, in gene pairs associating g1 with each other gene from the cisRED database (g2, g3, g4, g5. . . gn). Each (*l*, *d*)SSM is corrected by the number of potential SSMs as described in methods section (SSMC). Next, in order to evaluate the effect of atomic motif sizes in the evaluation of the number of potential SSMs, we computed the distribution of the number of SSMs for 50,000 pairs of randomly selected genes, showing that the number of SSMs found for gene pairs is correlated with the number of potential SSMs. Representative distribution for (6,0), (8,1), (10,2) and (14,4) SSM are displayed in Figure 3. These data demonstrate that the number of SSMs found for gene pairs is correlated with the number of potential SSMs thereby requiring a correcting factor leading to the SSMC. Then, the exceptional nature of each SSMC is measured by testing the null hypothesis that the selected gene pairs have a higher SSMC than expected by chance with random genes (using a SSMC empirical distribution computed from 50,000 pairs from the cisRED database). The probability of finding an equal or greater SSMC value than by chance, is used as an estimated *p*-value for the null-hypothesis. Next, the *p*-values are computed for each of the (*l*, *d*)SSMCs and the *cp*-value is defined as the lowest *p*-value, considered as exceptional if its value is less than a defined threshold *t*. Given a gene g1 and a list L of (*l*, *d*)SSMC genes, we finally identify the CEXlist(*g*1, *L*, *t*) as a subset of the cisRED genes sharing a Combined EXceptional (CEX) number of SSMs.

To investigate the relevance of CEXlists, we analyzed 31 genes (Table 1), 20 were randomly selected and 11 with known function were arbitrarily chosen and used as internal control for gene annotation analysis. Each gene (g1, g2. . . g31) was independently submitted to a SSM analysis versus all genes from the cisRED database leading to 31 independent analyses of 18.000 gene pairs. The 31 CEXlists, obtained with the SSM search, were independently submitted to both GSEA and Gemma analysis as described in the following.

**Table 1:** CEXlist test set

GENE SYMBOL	DESCRIPTION
APLP1	Amyloid beta (A4) precursor-like protein 1
C6orf62	HBV X-transactivated gene 12 protein
C9orf3	aminopeptidase O
CLK3	CDC-like kinase 3
DEFA3	Defensin, alpha 3, neutrophil-specific
DUSP12	Dual specificity phosphatase 12
EEF1D	Eukaryotic translation elongation factor 1 delta (guanine nucleotide exchange protein)
FSHR	Follicle stimulating hormone receptor
MNT	MAX binding protein
MRGPRF	MAS-related GPR, member F
SH3D19	SH3 domain protein D19
TRIM61	Putative tripartite motif-containing protein 61
C1orf216	chromosome 1 open reading frame 216
C2orf67	chromosome 2 open reading frame 67
OR51Q1	Olfactory receptor, family 51, subfamily Q, member 1
CCDC64B	Coiled-coil domain-containing protein 64B
SLC9A3R2	solute carrier family 9 isoform 3 regulator 2
SPG7	Spastic paraplegia protein 7
WISP2	WNT1 inducible signaling pathway protein 2
SNRPD2	Small nuclear ribonucleoprotein Sm D2 (snRNP core protein D2) (Sm-D2)
ADAM12	ADAM metallopeptidase domain 12 (meltrin alpha)
SMAD2	SMAD family member 2
SMAD3	SMAD family member 3
AURKA	Aurora kinase A
AURKB	Aurora kinase B
AURKC	Aurora kinase C
ACTA1	Actin, alpha 1, skeletal muscle
ALB	Albumin
ALDOA	aldolase A, fructose-bisphosphate
DES	Desmin
LRRTM1	Leucine rich repeat transmembrane neuronal 1

Twenty genes (lane 1 to 20) were randomly selected from the cisRED database. Eleven genes (lane 21 to 31) with known functions were arbitrarily added to complete the random selection and used as internal controls for gene annotation analyses.

### SSM patterns identify gene clusters associated with specific biological categories

**Figure 4:**
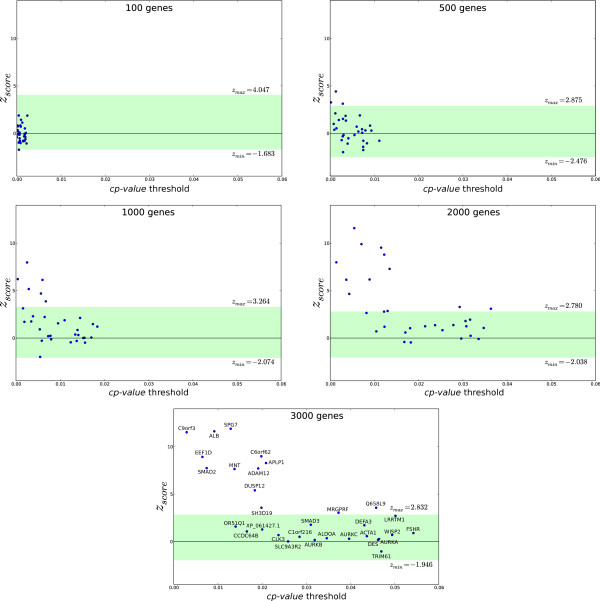
**Variation of the *z_score _*as a function of the *cp*-value threshold for various CEXlist sizes**. SSM analysis was applied to genes from the test set and non-specific variations were calculated for 200 random gene lists (green area).

To show that the SSM-based approach does indeed select genes that share putative common regulatory motifs with a gene of interest, we characterized genes in CEXlists using biological category annotations (Gene Ontology and KEGG pathways). Genes involved in the same biological process have a greater likelihood of being coordinately expressed, thereby potentially sharing co-regulation patterns. For this purpose, we investigated the over-representation of specific categories in CEXlists compared with random lists of gene. Briefly, CEXlists were computed for a test set of 31 genes (Table 1) and the 31 lists were further subjected to gene-set enrichment analysis. *p*-values for over-represented categories in CEXlists were computed to define the *c_scores _*that measures the relative importance of gene clustering in over-represented categories. Finally, *z_scores _*were calculated to compare the *c_scores _*obtained from different CEXlists with the distribution of randomly selected genes. As shown in Figure 4, the *z_scores _*varied according to the thresholds of defined sizes of CEXlist. Note that the number of CEXlists with a significant *z_scores _*strongly increased with the size of the tested lists including 3, 6, 11 and 13 CEXlists for lists of 500, 1000, 2000 and 3000 genes, respectively. This observation could be related to the nature of the *c_scores_*. Indeed, the *c_scores _*depends on both the *p*-value of over-represented categories and the number of categories which affects the number of terms in the sum $\overset{n}{\underset{i=1}{\sum }}\log\left(\frac{1}{p_{i}}-19\right)$. For small lists of genes, the number of over-represented categories is low and variation in the number of categories might affect the *c_scores_*, introducing some noise. Interestingly, we observed that CEXlists with low *cp*-value threshold showed higher *z_scores_*, suggesting that the *cp*-value threshold used for generating CEXlists is more relevant than the fixed CEXlist size (Figure 4). In addition, CEXlists which showed significant variation when compared to random lists were CEXlists obtained from genes characterized by specific regulatory profiles, *e.g. *genes expressed in differentiated tissue or highly induced by microenvironment stimulation (including the CEXlists obtained from ALB, ADAM12, SPG7 and C9orf3). We expect that genes with such specific expression should be characterized by the presence of strong specific regulatory motifs (such as the binding site for MyoD in all muscle-specific genes). In contrast, constitutively expressed housekeeping genes that show low regulatory patterns should exhibit weak signatures that are not considered significant by the SSM approach. In order to validate this hypothesis, a larger set of genes needs to be tested, however, out of the test set, our results assigned high scores to ALB (albumin), a marker for differentiated hepatocytes, and ADAM12, specific for the differentiation of mesenchymal cells (Figure 4, lower panel). Although ubiquitously expressed SPG7, a mitochondrial protease that belongs to the m-AAA protease family but displays specific substrates that affect mitochondrial biogenesis in a tissue-specific manner, was also selected. So was C9orf3 (aminopeptidase O), which has been described as playing a role in the proteolytic processing of bioactive peptides in specific tissues such as testis and heart. Genes with low z-scores included genes of unknown function such as TRIM61 (Putative tripartite motif-containing protein 61) or ubiquitously expressed genes such as SLC9A3R2, which encodes a scaffold protein that connects plasma membrane proteins with members of the ezrin/moesin/radixin family (Figure 4, lower panel).

In support of these findings, we show that the over-represented biological categories associated with a CEXlist indeed matches the functional specificity of the gene considered. For example the categories associated with the olfactory receptor OR51Q1 are *olfactory receptor activity *and *sensory perception of smell *(Table 2). Similarly overrepresented GO categories for MYOG, the muscle-specific transcription factor involved in myoblast differentiation, are *skeletal muscle fiber development and myoblast migration*. Finally, we focused on ADAM12, a transmembrane disintegrin and metalloproteinase involved in differentiation of mesenchymal cells, cell adhesion and growth-factor signaling [32,33]. Over-represented biological categories in the corresponding CEXlist matched known ADAM12 functions, including *intracellular, intracellular signaling cascade, fibroblast growth factor activity, focal adhesion *(Table 2). Notably, we also identified the over-represented biological category *positive regulation of neurogenesis*, suggesting that ADAM12 might be co-regulated with genes involved in neuronal processes. In agreement with this observation, several neuronal markers were recently described in the hepatic stellate cells that are also the major source of ADAM12 in the liver [34].

**Table 2:** Representative categories

CATEGORY	p-value
**OR51Q1**	
olfactory receptor activity (MF)	5.7 × 10^-10^
sensory preception of smell (BP)	2.4 × 10^-10^
**MYOG**	
skeletal muscle fiber development (BP)	3.8 × 10^-3^
myoblast migration (BP)	4.8 × 10^-3^
**ADAM12**	
fibroblast growth factor activity (MF)	1.2 × 10^-3^
intracellular signaling cascade (BP)	1.8 × 10^-3^
intracellular (CC)	1.7 × 10^-7^
focal adhesion (K)	6.1 × 10^-3^
positive regulation of neurogenesis (BP)	2.5 × 10^-4^

Representative categories in CEXlists from OR51Q1, MYOG and ADAM12. MF, Molecular Function; BP, Biological Process; CC, Cellular Component and K, KEGG pathways.

Taken together, our data clearly demonstrate that the SSM approach identifies lists of genes, which are significantly more closely associated within given biological processes than randomly selected lists of gene. In addition, the over-represented categories verify known function of the genes and also allow the prediction of new ones. Finally theses results suggest that SSMs select genes that share similar regulatory patterns.

### Co-expression is a significant feature of genes identified by the SSM approach

**Figure 5:**
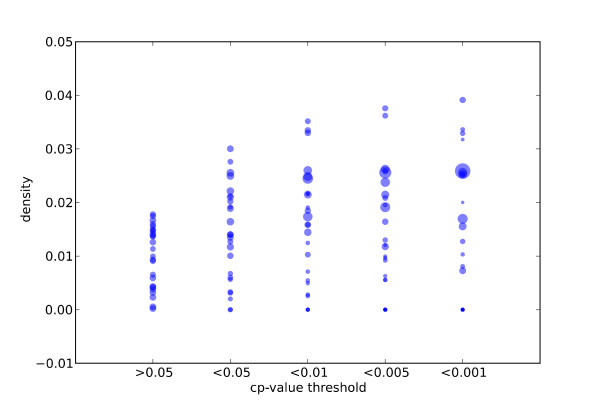
**Association between SSM enrichment and co-expression**. Genes (circles) from the sample set were submitted to both SSM and Gemma analyses and the overlap between genes was expressed as a density value (number of Gemma genes per gene in CEXlist) according to different *cp*-value thresholds. Circle size is correlated with the number of genes in CEXlists.

Co-expression criteria, widely used to search for common regulatory elements among genes and high throughput transcriptome data, now provides an important biological resource. Although gene co-expression does not imply similar regulation, especially when tissue transcriptomes are investigated, we hypothesized that genes selected in CEXlists might be co-transcribed in specific biological contexts. We took advantage of Gemma, a database and software suite for the meta-analysis of gene expression data. Gemma contains 1474 array experiments, including 584 human data sets, which we screened with genes from our test set (Table 1). We compared the genes obtained from SSM analysis (CEXlists) to those obtained from Gemma analysis (co-expressed genes). Briefly, each gene from the test set was used as input for both SSM analyses and Gemma (co-expression search, the scope was set to all human and the stringency to 3, results were limited to 500 genes containing in priority genes more often observed co-expressed with the gene of interest), note that, at the time the analysis was performed, Gemma contained data for 25 genes of the 31 genes test set. The overlap between genes issued from Gemma and SSM analyses was expressed as a density value, the number of Gemma genes per gene belonging to the CEXlist. As shown in Figure 5, density was clearly correlated with the *cp*-value threshold. This suggests that genes sharing high numbers of SSMs with another gene are significantly more often co-expressed with this gene than genes sharing lower numbers of SSMs. Finally, we used Fisher tests to compare Gemma genes among genes in CEXlists (*cp*-value <*t*) to Gemma genes among genes out of CEXlists (*cp*-value >*t*). This comparative analysis was performed using 25 genes and 4 *cp*-value thresholds leading to 100 samples. 61 samples show an enrichment of co-expressed genes in CEXlists, Fisher tests identified 29 significant cases (*p*-value < 0.05) - and one significant decrease. Results are presented in Table 3. This analysis lends further support to our conclusion that CEXlists are enriched in co-expressed genes.

**Table 3:** Comparison between co-expressed genes and not co-expressed genes in CEXlists.

*cp*-value threshold	0.05	0.01	0.005	0.001
ACTA1	1.601	1.168	1.187	2.215
DES	1.366*	1.285	0.659	0.000
SPG7_HUMAN	2.078*	1.957*	1.846*	2.078*
SMAD2	1.877*	1.414*	1.297	1.361
SMAD3	1.363*	1.354	1.206	0.000
ADAM12	0.803	0.965	1.003	0.777
C9orf3	1.662*	1.400*	1.443*	1.381
APLP1	0.879	1.228	1.380	0.525
WISP2	1.311	0.559	0.000	0.000
SLC9A3R2	1.079	1.083	1.380	2.673
MNT	1.789*	1.893*	1.426	0.855
ALDOA_HUMAN	1.008	0.986	0.759	0.000
DEFA3	0.974	1.576	0.000	0.000
AURKA	1.274	1.247	0.687	1.145
AURKB	1.999*	2.263*	2.249*	2.353*
FSHR	0.000	0.000	0.000	0.000
LRRTM1	0.871	0.000	0.000	0.000
AURKC_HUMAN	0.556*	0.515	1.180	0.000
MRGPRF	1.559	1.697	1.300	0.000
Q658L9_HUMAN	0.000	0.000	0.000	0.000
DUSP12	1.446*	2.139*	1.555	1.993
EEF1D	1.649*	1.515*	1.634*	1.348
ALB	1.953*	1.877*	1.769*	0.972
C6orf62	1.930*	1.756*	1.926*	1.626
CLK3	0.759*	0.970	0.558	1.953

For four *cp*-value thresholds, *t*, the table compares Gemma genes present in CEXlists (*cp*-value <*t*) and Gemma genes not present in CEXlists (*cp*-*value *>*t*). For genes present or not in CEXlists, the ratio of genes in Gemma per gene is computed. The final value in the Table is the ratio of the result obtained for a CEXlist and the genes out of it. Thus, a value above one indicates an enrichment of co-expressed genes in a CEXlist. Fisher tests were used to compare the distribution of Gemma genes in and out of CEXlists, a "*" indicates a distribution significantly different (*p *< 0.05).

### Genes involved in a same biological process or over-expressed in a same tissue show higher SSM numbers

To confirm that SSM analyses capture regulatory motif patterns, we compared the SSM number for genes either involved in the same biological pathway or over-expressed in the same tissue, two conditions that have been suggested to implicate putative co-regulatory processes. For this purpose, we selected 2539 genes distributed in 162 different human pathways from the KEGG database and 1228 genes specifically expressed in 28 different tissues from the TIGER database.

We hypothesized that genes implicated in the same pathway or over-expressed in the same tissue may share more common regulatory mechanisms than random genes. To test this, we extensively computed the most exceptional number of SSMs (*cp*-value) for every pair of genes involved in the same pathway or tissue. These results were then compared to the *cp*-values computed for a sample of randomly selected pairs of genes extracted from different pathways or tissues. The samples contained 70,000 and 100,000 pairs of genes for the KEGG and TIGER databases, respectively.

As shown in Table 4, the number of pairs with an exceptional SSM number was significantly higher between pairs of genes involved in the same biological pathway or expressed in the same tissue than in different ones. It is important to note that the increase in *cp*-value threshold stringency was associated with an increase in enrichment of gene pairs selected within a biological process (KEGG) or tissue (TIGER), with a maximum of a 3.484-fold increase for *cp*-value = 10*e*^-5^. Among tissues, placenta had the highest SSM-based pairs of genes, including PSG1/PSG6, PSG1/PSG11, PSG1/PSG8, PSG1/PSG9 and PSG8/PSG9 pairs, which showed highly significant *cp*-values (< 8.10^-5^). It is of interest to note that the common regulation between members of PSG family genes has been suggested to be related to chromatin structure [35], suggesting that epigenetic markers might be also detected by the SSM approach. Taken together, our data show that genes associated with the same biological process or expressed in the same tissue share more SSMs than random genes, thereby serving as a useful and novel marker for common regulatory mechanisms.

**Table 4:** Comparative analysis of SSM counts with TIGER and KEGG databases

Threshold	KEGG	TIGER
0.01	1.081*	1.117*
0.005	1.087*	1.134*
0.001	1.094*	1.115
0.0001	1.197	1.234
0.00001	1.404	3.484*

Analysis of SSM count for genes expressed within specific tissues (TIGER database) or pathways (KEGG database). According to different thresholds, the number of gene pairs with an exceptional SSM count divided by total gene pairs was computed for genes either associated or not associated, within the same tissue or pathway. Data are expressed as a ratio between these two values, a ratio superior to 1 indicating an enrichment of gene pairs with an exceptional SSM number when genes are expressed in a same tissue or pathway(*, p ≤ 0.05)

## Discussion

Many methods have been developed to identify *cis*-regulatory elements and the recent integration of both phylogenetic footprinting and co-expression data demonstrably enriches the predictive function of these methods. However, our understanding of the regulation of gene expression is far from complete and the discovery of functionally important sequence modules remains a difficult task. To improve on these *in silico *investigative methods, instead of searching for putative regulatory motifs [36-38], we searched for genes which share common sequence profiles in promoter regions, without *a priori *information about sequence motifs *per se*. In agreement with our approach, others have shown that regulatory signals are supported by the involvement of combinatorial interactions between transcription factors that function as *cis*-regulatory modules with complex signatures [39] and dynamics [40]. We based our method on previously computed conserved sequences, using the atomic motifs from the cisRED human database. Other methods can be used to identify conserved sequences such as the global multiple alignment [41] and Footprinter approaches [42]. Note that conserved upstream sequences from the CORG database [43] have been previously used to search for short regulatory motifs [44]. However, CORG only includes orthologous genes from the human and mouse genomes and the authors further reduced their benchmark by adding filters based on common Gene Ontology and Gene Expression. In the present study, our analysis was conducted on more than 18,000 conserved sequences across 40 species and all pairs of genes implicating a gene of interest were exhaustively investigated.

The performance of the SSM approach to uncover gene expression regulation signatures is demonstrated by the SSM enrichment of genes co-expressed in similar tissues (TIGER) or biological pathway (KEGG). An originality of the method is the creation of CEXlists which are lists of genes predicted to be co-regulated with a gene of interest. We demonstrated that the genes obtained in a CEXlists for a gene *g*, show clustering in biological categories related to the function of *g *and are significantly more co-expressed with *g *than randomly selected genes.

It is important to note that although we have shown the SSM approach was able to uncover regulatory signals, the fuzzy nature of SSM makes difficult to relate specific results to previously described regulatory signals such as TFBS. This is especially true when the result is obtained with short and degenerated SSM (in the case of (6, 2)SSM for instance).

During last years, numerous integrative approaches that search for regulatory elements have been developed by incorporating co-expression datasets and/or ontology annotation within an unique algorithm to improve the discovery of regulatory modules in various organisms such as archeae [45], bacteria [46], yeast [47] and human [48,49].

Overall these approaches are thought to be promising, computational predictions are still mainly based on genomic sequences [50]. However, one criticism that can be formulated regarding current sequence-based methods (including ours) is that they do not yet take into account the microenvironmental regulatory context, including epigenetic factors and the dynamics of transcription factor binding, although these sequences necessarily share regulatory signals. Such epigenetic signals might be captured using fuzzy sequence based method such as SSM. Nevertheless, integration of additional biological information linked to gene regulation, including methylation and chromatin remodeling might improve cis-regulatory patterns discovery in the future.

## Conclusions

The coordinated transcriptional regulation of gene expression is essential for cells to respond to their environment and mediate complex processes including proliferation, differentiation and death. Binding of transcription factors to cognate DNA binding sites within promoters of genes can account for their expression and numerous methods have been developed to identify or predict transcription-factor binding sites. However, data derived from genome-wide sequencing, high-throughput analyses of DNA-protein interactions and integration of epigenetic signaling necessarily lead to a much more intricate view of the mechanisms that account for the regulated expression of gene networks. Accordingly, a major challenge lies in the development of new computational approaches that successfully extract from DNA sequence alone gene expression signatures characteristic of ensembles of co-regulated or co-expressed genes. The new approach we describe clusters genes from whole-genome sequences according to a broad range of degenerate shared short sequence motifs. This successfully selects for genes that are highly enriched for sets associated with given biological processes or found to be significantly more frequently co-expressed in the same tissues. The computational identification of genes that possess such functional signatures should prove useful to decipher the multi-layered patterns of co-regulated gene expression that form the basis for complex biological pathways.

## Authors' contributions

JG conceived the study, performed the computational analyses and drafted the manuscript. ML conceived the study and drafted the manuscript. NL conceived the study and drafted the manuscript. NT conceived the study and drafted the manuscript. All authors read and approved the final manuscript.

## Supplementary Material

Additional file 1 **Diagram of the algorithm leading to the construction of a set of (l, d)SSM for a pair of genes**. A) Representation of the atomic motifs for 2 genes (gray). Gene 1 and Gene 2 have 3 and 2 atomic motifs, respectively. The colored areas stress some subsequences of length *l *of the atomic motifs used as examples in the following panels, matching colors indicate matching extended sequences. In the first step of the algorithm, a sliding window of length *l *travels through the sequence of all atomic motifs, analyzing all overlapping subsequences. B) Subsequences drawn from the first step of the algorithm are stored in PreSSM structures. 2 subsequences having matching extended sequence are stored in the same PreSSM (*i.e. *PreSSM 2). PreSSMs store the identifier of the genes the subsequences were drawn from. C) A graph is created whose nodes are PreSSMs and vertices between 2 nodes indicate a Hamming distance ≤ *d *between the extended sequence of 2 PreSSMs. Maximal cliques of the graph are computed. D) Maximal cliques whose PreSSMs contain subsequences from genes 1 and 2 are (*l*, *d*)SSM. In the diagram example, 2 SSMs are found. Click here for file
