# Supplementary material for: Simple Shared Motifs (SSM) in conserved region of promoters: a new approach to identify co-regulation patterns
Source: BMC Bioinformatics. 2011 Sep 12;12:365. doi: 10.1186/1471-2105-12-365 (PMC3215511; doi:10.1186/1471-2105-12-365)
Supplement: Additional file 1 — Diagram of the algorithm leading to the construction of a set of (l, d)SSM for a pair of genes. A) Representation of the atomic motifs for 2 genes (gray). Gene 1 and Gene 2 have 3 and 2 atomic motifs, respectively. The colored areas stress some subsequences of length l of the atomic motifs used as examples in the following panels, matching colors indicate matching extended sequences. In the first step of the algorithm, a sliding window of length l travels through the sequence of all atomic motifs, analyzing all overlapping subsequences. B) Subsequences drawn from the first step of the algorithm are stored in PreSSM structures. 2 subsequences having matching extended sequence are stored in the same PreSSM (i.e. PreSSM 2). PreSSMs store the identifier of the genes the subsequences were drawn from. C) A graph is created whose nodes are PreSSMs and vertices between 2 nodes indicate a Hamming distance ≤ d between the extended sequence of 2 PreSSMs. Maximal cliques of the graph are computed. D) Maximal cliques whose PreSSMs contain subsequences from genes 1 and 2 are (l, d)SSM. In the diagram example, 2 SSMs are found. [file 1471-2105-12-365-S1.pdf]

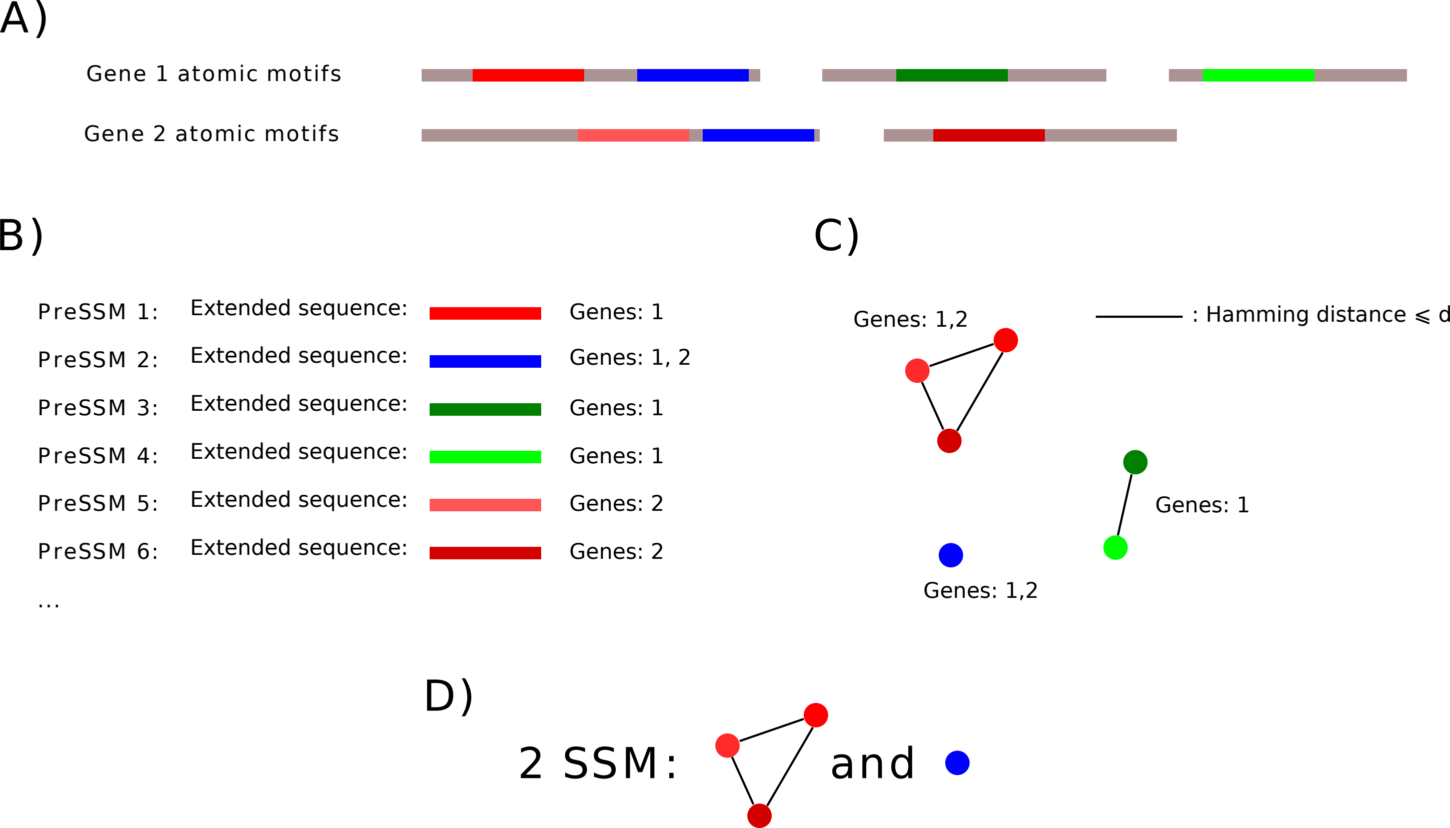
A)
Gene 1 atomic motifs
Gene 2 atomic motifs
B)
PreSSM 1: Extended sequence: Genes: 1
PreSSM 2: Extended sequence: Genes: 1, 2
PreSSM 3: Extended sequence: Genes: 1
PreSSM 4: Extended sequence: Genes: 1
PreSSM 5: Extended sequence: Genes: 2
PreSSM 6: Extended sequence: Genes: 2
...
C)
Genes: 1,2
: Hamming distance ⩽ d
Genes: 1
Genes: 1,2
D)
2 SSM:
and
